# Partial unilateral ovarian torsion in a red-eared slider turtle (*Trachemys scripta elegans*)

**DOI:** 10.3389/fvets.2025.1524568

**Published:** 2025-02-05

**Authors:** Alessandro Vetere, Martina Gavezzoli, Lucia Victoria Bel, Rosanna Di Lecce, Martina Fumeo, Mattia Bonazzi, Francesco Di Ianni

**Affiliations:** ^1^Department of Veterinary Science, Università Degli Studi di Parma, Parma, Italy; ^2^New Companion Animals Veterinary Clinic, Faculty of Veterinary Medicine, University of Agricultural Sciences and Veterinary Medicine Cluj-Napoca, Cluj-Napoca, Romania

**Keywords:** ovarian torsion, follicular torsion, *Trachemys scripta elegans*, reproductive disorders, reptiles, Chelonia

## Abstract

Follicular torsion occurs when only a group of ovarian follicles rotates around its own axis resulting in vascular compromise. To our knowledge, no previous reports have documented the occurrence of this condition in chelonians. A 14-year-old female *Trachemys scripta* was presented with inappetence and lethargy for approximately 2 weeks. Diagnostic tests, including complete blood work, radiography, and ultrasound, were performed. Radiographs showed no pathological changes, while blood tests showed mild heterophilia. Ultrasound revealed multiple, round, heterogeneous hyperechoic follicles and free anechoic fluid in the coelom. The color flow examination through the right pre-femoral fossa revealed the absence of blood flow in a group of follicles. A total body CT scan highlighted several rounded formations, four of which contained disomogeneus areas. Fluid with an air-fluid level and gas-filled areas were also noted in the coelomic cavity. A diagnosis of preovulatory follicular stasis and coelomitis was made, and a bilateral ovariosalpingectomy was performed endoscopically via the right prefemoral fossa after 24 h stabilization. There was a 360° torsion in a group of follicles, which appeared dark and had an increased consistency compared to adjacent follicles. Adhesions between the pathological follicles and surrounding tissue were observed. Bacteriological analysis of the coelomic fluid revealed the presence of *Klebsiella* spp. Histopathological examination of both ovaries showed coagulative necrosis, hemorrhage, congestion, and vascular thrombosis, along with a mixed inflammatory infiltrate. Post-surgical treatment with marbofloxacin and meloxicam resulted in significant clinical improvement. The animal was discharged 15 days after surgery, with a normal appetite.

## Introduction

Ovarian torsion is a serious medical condition that occurs when an ovary twists around the around its axis, compromising blood flow. This can lead to reduced venous return, stromal edema, internal hemorrhage, and infarction, along with subsequent complications. Follicular torsion occurs when only a group of ovarian follicles rotate around its own axis. The torsion can be complete (360°) or partial (1–3).

Ovarian torsion in reptiles is rarely reported in the literature (1–5). To date, there are no reports in the order Chelonia. Sporadic cases have been reported in two species of iguana (*Iguana iguana* and *Cyclura cornuta*) (2), in a panther chameleon (*Furcifer pardalis*) (3), and in an Moroccan eyed lizard (*Timon tangitanus*) (1). Only one case of follicular torsion has been reported in literature, involving an iguana (*Iguana iguana*) (4). The clinical presentation was defined by non-specific symptoms, like anorexia, lethargy, and signs of pain-related behavior. In two cases surgery was performed and the animal recovered quickly (2, 3). Otherwise, the diagnosis was made postmortem. In iguanas, comorbidities affecting the reproductive system, such as bacterial oophoritis and egg retention, have been documented (2).

In a recently published paper, five cases of ovarian torsion were diagnosed in five geckos. All animals underwent to a bilateral ovariosalpingectomy. Surgery was resolutive in all but one gecko, that died few hours post operatively (5).

To date, in birds, only two reports of follicular torsion were published: the first case involves a young ostrich and the second in an older penguin. In both cases, the animals exhibited acute or hyperacute symptoms, dying, respectively, after 3 days and 1 day from the onset of symptoms. Both animals showed signs consistent with acute abdominal pain, anorexia, and significant depression. In both cases, the diagnosis was made post-mortem (6, 7).

In mammals, only case reports of ovarian torsions are currently documented. Three of these are associated with comorbidities or paraphysiological conditions of the reproductive system (pyometra, pregnancy, suspected neoplasia), and one is reported in a foal.

One case involved a 2-year-old female Golden Retriever with a pyometradiagnosis. A complete torsion of the left ovary was found duringovariohysterectomy (8).

In another report, a 8-month-old, female Husky developed ovarian torsion due to the torsion of the gravid uterine horn and uterine rupture (9). In a rabbit, the torsion was diagnosed during a routine check-up as the animal was completely asymptomatic. The causes of the torsion were not completely clarified, but a previous ovarian neoplasm is suspected, which may have led to the enlargement of the ovary and the subsequent torsion (10).

A 720-degree torsion of the left mesovarium was found in a 1-week-old foal that presented with acute abdominal pain. The affected ovary was surgically removed, and the foal fully recovered, showing no complications 2 years after the surgery (11).

In all these cases, the condition was treated surgically. Only in the case of the rabbit was the animal asymptomatic, while the other animals exhibited symptoms consistent with an acute abdomen.

In human medicine, ovarian torsion accounts for approximately 2–3% of gynecological emergencies (12). It is reported across all age groups, from pediatric patients to menopausal patients, but it is more common in patients of reproductive age (13). Torsion is reported to be associated with ovarian cysts or masses, typically larger than 5 cm in diameter. However, it can also occur in healthy ovaries, especially in prepubescent girls due to the presence of more elastic and elongated infundibulopelvic ligaments (12). Early diagnosis is crucial and often relies on transvaginal ultrasound and computed tomography (CT) scans (14). The primary treatment is emergency surgery to untwist the ovary and restore blood flow, with the goal of preserving the organ. This approach is particularly recommended for prepubescent patients, and oophoropexy is performed to prevent recurrence. Several studies show that timely intervention significantly increases the chances of preserving ovarian function into adulthood. If not treated promptly, ovarian torsion can lead to ovarian necrosis, resulting in the loss of the ovary and potential future fertility complications (12). The situation is different when a neoplastic process is suspected as the cause of the torsion; in this case, unilateral ovariectomy is indicated.

### Case description

A 14-year-old female pet *Trachemys scripta* weighing 2.1 kg with a curved carapace length (CCL) of 22 cm was presented to a veterinary hospital in Parma due to anorexia and lethargy lasting approximately 2 weeks. The animal was kept indoors in a glass aquarium with a UVB 5.0 spectrum light and with no heating sources. Temperature was never monitored. The food consisted of commercial food (shrimp) and occasional vegetables. The owner did not report any history of egg-laying. Upon clinical examination, the animal appeared in a good nutritional status, but it was lethargic.

Complete hematological exams, X-rays, and an ultrasound examination were performed.

A 1 mL sample of venous blood was drawn from the jugular vein for a complete blood cell (CBC) count and biochemical analysis, demonstrating a slight heterophilia (68%) [Normal Value: 7–65%] compared with the published reference values for the species (15, 16).

Three radiograph projections (latero-lateral, dorsal-ventral, cranio-caudal) were performed. No pathological findings were noted.

A coelomic ultrasound examination was performed through the left and right prefemoral fossa using a microconvex probe. The presence of multiple heterogeneous hyperechoic follicles (ranging in size from 1 to 2 cm) was noted. Anechoic free fluid was found in the coelom. Hyperechoic foci with posterior comet tail artifacts were seen free in the coelomic cavity and within the fluid material revealing the presence of free gas.

**Figure 1:**
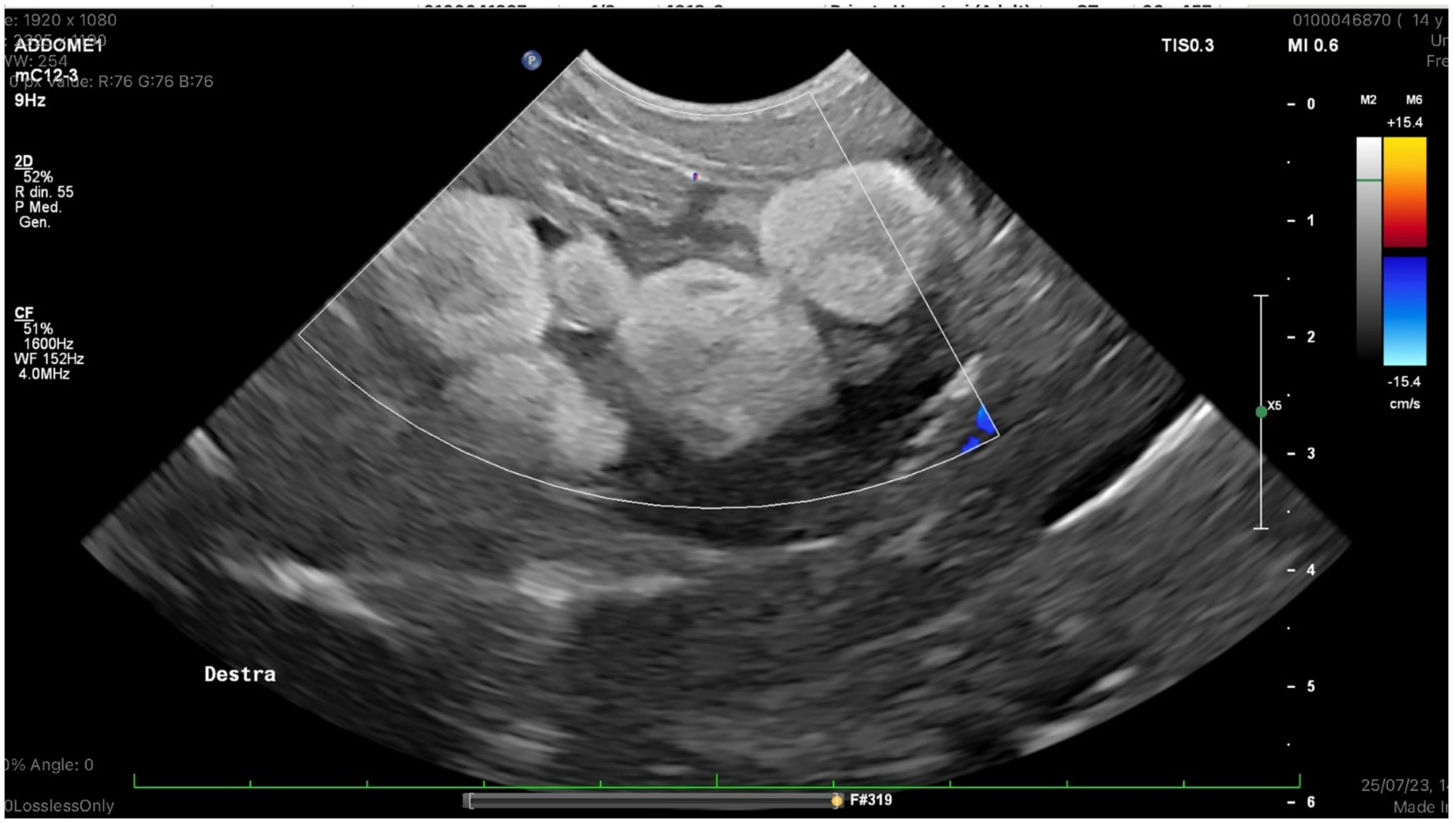
Right ovary, color doppler examination: No ovarian blood flow was detected in a group of follicoles.

The color flow examination through the right pre-femoral fossa revealed the absence of blood flow in a group of follicles (Figure 1).

**Figure 2:**
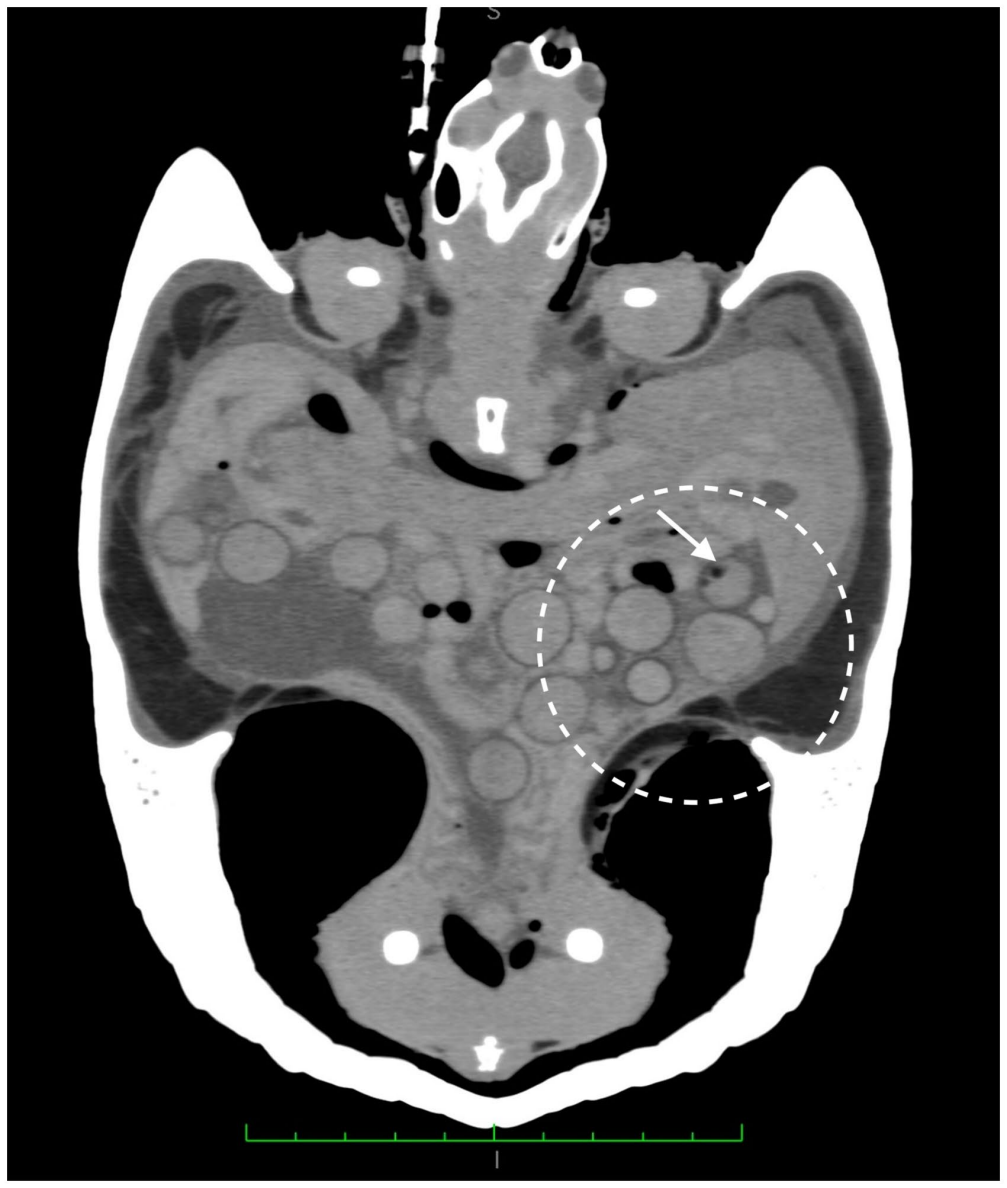
CT scan, numerous rounded formations, showing dysomogeneous areas and gas bubble (white arrow). Hyperattenuating fluid material with an air-fluid level free in the coelomic cavity and gas-filled formations resembling gas bubbles free in the coelomic cavity between the organs are visible. Coronal plane.

A CT scan was performed for a better investigation of the coelomic organs. The animal was sedated with Alfaxalone (Alfaxan multidose 10 mg/mL, Zoetis Italia s.r.l. Via Andrea Doria, 41 M 00192 Rome, Italy) at 10 mg/kg intramuscularly. An intravenous (IV) 26 gauge Teflon™ catheter (Deltaven S.p.A. Via dell’Industria, 120094 Corsico (MI), Italy) was placed in the right jugular vein and a iodinated contrast medium at 2 mL/kg (ioversol [300 mg/mL]; Optiray 300; Guebert SpA Via Aurelio Saffi, 920,090 Segrate (MI), Italy) was administered. During the contrast agent injection, a vein rupture occurred, causing the contrast agent to leak into the surrounding connective tissue. It was not possible to reposition another venous access. The CT scan revealed numerous rounded formations, four of which had dysomogeneous areas. The scan also showed hyperattenuating fluid material with an air-fluid level Hounfield Units (HU) 12 free in the coelomic cavity, and gas-filled formations resembling gas bubbles free in the coelomic cavity between the organs (Figure 2).

A diagnosis of coelomitis associated with preovulatory follicular stasis was made. The lack of follicle perfusion observed with color Doppler on the right did not rule out the possibility of ovarian or follicular torsion. The animal was stabilized with fluid therapy with Lactated Ringer’s at 30 mg/kg/die SC (Ringer Lattato 500 mL Salf S.p.A. Via Aldo Moro, 420,090 Cernusco sul Naviglio (MI), Italy), analgesic therapy with tramadol IM at 10 mg/kg q72h (Altadol 50 mg/mL, Formevet S.r.l. Via Orsini, 1,520,123 Milan, Italy), and antibiotic therapy with marbofloxacin IM at 2 mg/kg q24h (Xeden iniettabile 50 mg/mL Ceva salute animale S.p.A. Via Colleoni, 1,520,041 Agrate Brianza (MB), Italy). Surgery was scheduled for the following day.

The subject was premedicated with ketamine at 10 mg/kg IM, (Ketavet 100,100 mg/mL, MSD Animal Health S.r.l. Via Fratelli Cervi, 720,090 Segrate (MI), Italy) dexmedetomidine at 50 μg/kg IM (DEXDOMITOR 0.1 mg/mL soluzione iniettabile, Zoetis Italia s.r.l. Via Andrea Doria, 41 M 00192 Rome, Italy) and midazolam at 0.7 mg/kg IM (Hameln Pharmaceuticals S.r.l. Via Ariberto, 1,620,123 Milan, Italy). An intravenous (IV) 26 gauge Teflon™ catheter (Deltaven S.p.A., Via dell’Industria, 1, 20,094 Corsico (MI), Italy) was placed in the left jugular vein in order to administer propofol (PropoVet Multidose 10 mg/mL Zoetis Italia s.r.l. Via Andrea Doria, 41 M 00192 Rome, Italy) as needed, up to a maximum dosage of 5 mg/kg EV. The animalwas monitored using Doppler sound (Doppler VET BP Sonomed S.p.A. Via Carlo Cattaneo, 1,620,092 Cinisello Balsamo (MI), Italy), ECG and capnography (Monitor ePM 12 Vet Mindray, Mindray Mindray Building, 21st Century Tower, No. 9, Keji 12th Road, Hi-tech Zone, Shenzhen, Guangdong, 518,057, China) to assess the depth of anesthesia. The animal was subsequently intubated using a 20 gauge Teflon™ catheter (Deltaven S.p.A.Via dell’Industria, 120,094 Corsico (MI), Italy), was maintained under anesthesia with isoflurane at 1.5% (Iso-Vet Isoflurano 1,000 Mg/mL, Piramal Critical Care Italia S.p.A. Via Bologna, 2,820,093 Cologno Monzese (MI), Italy) and was manually ventilated with a 0.5-liter bag at a rate of 4 breaths per minute. A bilateral ovariosalpingectomy was performed using endoscopic assistance via the right pre-femoral fossa.

The animal was positioned in left lateral recumbency and secured with two positioning sandbags to prevent sliding. The hind limbs were extended and taped together with adhesive surgical tape to improve access to the pre-femoral fossa. The right pre-femoral fossa and adjacent shell were aseptically prepared with a 2.0% chlorhexidine digluconate solution (Clorexinal 2%, Nuova Farmec, Via Walther Fleming 7, Settimo, VR, Italy).

A transparent surgical drape was then applied to ensure the surgical site remained aseptic. A skin incision of approximately 2 cm was made in a craniocaudal direction using a no. 11 surgical blade (Mealli srl, Borgo Santi Apostoli, Firenze, Italy). Blunt dissection followed to separate the subcutaneous tissues, revealing the aponeurosis of the ventral and oblique abdominal muscles. The muscles and coelomic membrane were then carefully dissected with the tip of mosquito forceps to enter the coelomic cavity, thus minimizing the risk of damaging the urinary bladder.

The presence of free clear fluid was observed upon opening the coelomic cavity. A 1 mL of fluid was aseptically sampled and sent to the laboratory for bacteriological examination.

Using a 2.7 mm × 18 cm, 30° oblique telescope with a 4.8 mm operative sheath (Storz Telepack TP100 EN, Karl Storz Endoscopia Italia Srl, Verona, Italy), the right ovary and oviduct were located and exteriorized with sterile swabs.

The ovarian pedicle, mesovarium, and mesosalpinx vessels were cauterized and excised with an electrocoagulation device (EnSeal®, Alcyon Italia, Via Nicotera 29, 00195 Roma). The oviduct was tied off approximately 3 cm from its cloacal opening using 2/0 absorbable monofilament suture (Monosyn® Braun Avitum Italy S.p.A. Mirandola, Italy) and then removed.

The coelom was inspected before closure to exclude the presence of residual ovarian tissue. The muscle layers were sutured with 2/0 absorbable monofilament (Monosyn® Braun Avitum Italy S.p.A. Mirandola, Italy) in a simple continuous pattern, and the skin was closed with everted simple interrupted sutures using the same material. The same procedure was performed on the contralateral ovary using the left prefemoral fossa for surgical access.

At the end of the procedurere it was reversed with atipamezole (Antisedan 5 mg/mL Vétoquinol Italia S.r.l. Via Cesare Pavese, 4,020,090 Segrate (MI), Italy) at a dose of 0.5 mg/Kg IM. A thorough visual inspection was performed once the ovaries were removed from the coelomic cavity.

**Figure 3:**
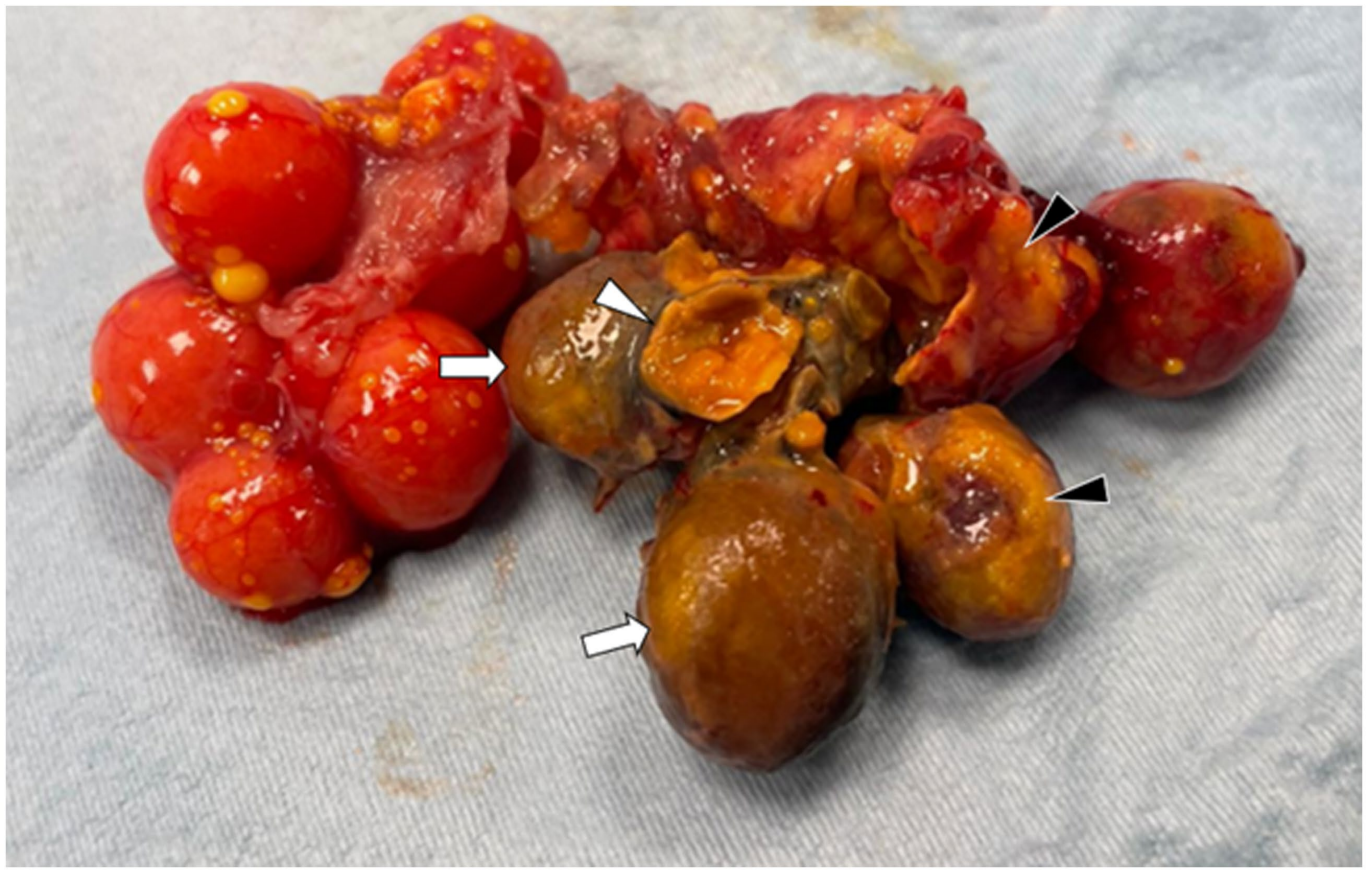
The right ovary shows a 360° torsion around the ovarian stalk (white arrows). The necrotic follicles are deformed, exhibiting irregular surfaces and a brown-yellow discoloration, with multifocally disrupted capsules (white arrowhead) and interfollicular adhesions. Ruptured follicles are partially covered by yolk material mixed with fibrin (black arrowhead).

The left ovary appeared grossly normal, while the right ovary showed a clear 360° torsion of a group of follicles (*n* = 4) around their vascular axis (Figure 3).

The affected follicles had a dark color and increased consistency compared to the adjacent follicles and the contralateral ovary. Additionally, numerous adhesions between the pathological follicles and the surrounding ovarian tissue were observed.

Meloxicam (Meloxidyl 5 mg/mL Ceva Salute Animale S.p.A. Via Colleoni, 1,520,041 Agrate Brianza (MB), Italy) at 0.4 mg/kg IM q24 for 5 days, along with marbofloxacin (Xeden 50 mg/mL Ceva salute animale S.p.A. Via Colleoni, 1,520,041 Agrate Brianza (MB), Italy) at 2 mg/kg IM q24 for 15 days and fluid therapy with Lactated Ringer’s at 30 mg/kg/die SC (Ringer Lattato 500 mL Salf S.p.A. Via Aldo Moro, 420,090 Cernusco sul Naviglio (MI), Italy) were administered postoperatively (17).

A noticeable improvement in the animal’s condition was observed 72 h postoperatively. The animal resumed eating on its own 2 weeks after the procedure and was then discharged. Bacteriology revealed the presence of *Klebsiella* spp. which was sensitive to marbofloxacin and intermediate to enrofloxacin.

**Figure 4:**
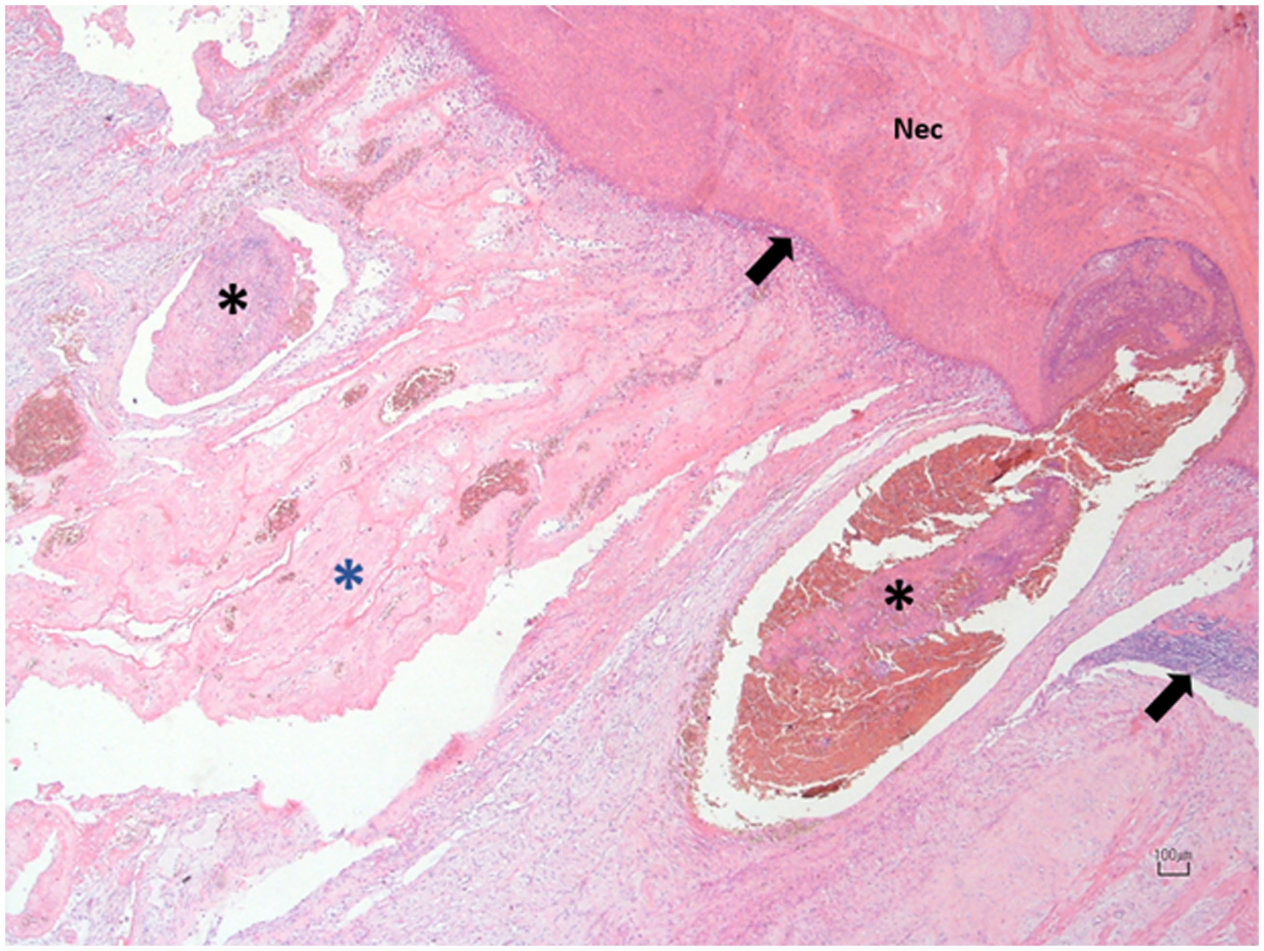
Photomicrograph of the right ovary at the site of torsion. The affected follicles exhibit extensive necrosis (N) with hemorrhagic replacement, bordered by a thin layer of mixed leukocytic infiltrate (black arrows). The adjacent blood vessels are congested and display prominent intraluminal thrombosis (black asterisks) and mural vasculitis. The surrounding ovarian tissue is markedly congested and distended by fibrin, admixed with blood, and yolk, and infiltrated by a few leukocytes (blue asterisk). Hematoxylin and Eosin; 10X. Scale bar: 100 μm.

Both the right and left ovaries were subjected to histopathological examination. The group of follicles affected by torsion and part of the adjacent parenchyma showed alterations consistent with coagulative necrosis, hemorrhagic areas, congestion and vascular thrombosis. Additionally, a mixed inflammatory infiltrate was observed within the organ’s parenchyma (Figure 4). The left ovary appeared normal.

A month after surgery, the animal was re-evaluated for a follow-up. The clinical examination showed no abnormalities, and the animal appeared alert, active, and had a normal appetite.

## Discussion

Reproductive disorders are frequently observed in captive reptiles (18). The most frequent conditions include infertility or failure to conceive, ovarian stasis, and egg retention (dystocia) (18). The causes of reproductive disorders in female chelonians in captivity are complex, multifactorial, and not yet fully understood (19). However, many authors suggest that poor management during the breeding season plays a significant role. Factors such as incorrect male-to-female ratios, improper or lack of male introduction, inadequate housing, absence of appropriate substrate for egg-laying, incorrect temperature and humidity ranges, and insufficient calcium supplementation and nutrition are commonly cited as contributors to the development of these disorders (19). In addition to environmental and management factors, the pathogenesis of these reproductive disorders is often influenced by concurrent medical conditions (19). Furthermore, pathological conditions directly involving the reproductive are also critical contributors to the development of these disorders (19).

In chelonians, reproductive disorders typically do not present with hyperacute clinical signs. More commonly, affected animals are brought in for evaluation due to a recent onset of dysorexia or anorexia, often occurring within the past week, along with signs of lethargy (18). These nonspecific symptoms, can be misinterpreted as normal by the owner during physiological conditions such as the oviposition period. To date, only one case of follicular torsion has been reported in reptiles, specifically in an iguana (4). All other case reports in reptiles, birds, and mammals refer to ovarian torsions. It is possible that the anatomy of the reptilian ovary, characterized by the presence of numerous follicles of varying sizes, could predispose them to developing follicular torsions as well. However, pathogenesis leading to a follicular torsion rather than an ovarian torsion remains unclear based solely on the single case available in the literature. In reports concerning ovarian torsion in lizards, the presence of painful symptoms was described (1–3); however, these signs are not easily recognized in chelonians.

The mild heterophilia noted in the complete blood count (CBC) is regarded as a nonspecific finding. In reptiles, heterophilia is commonly linked to inflammatory processes, which can arise from infectious diseases (such as bacterial and parasitic infections), tissue damage, and necrosis (16). The radiographic evaluation did not identify any abnormalities that could inform a diagnostic suspicion; however, ultrasound revealed alterations consistent with preovulatory follicular stasis. The lack of blood flow observed during the Color Flow examination could not rule out the possibility of a suspected follicular torsion affecting a cluster of follicles, although this could not be definitively assessed using this method. Considering the presence of this diagnostic suspicion, a contrast-enhanced CT scan was conducted. Unfortunately, the leakage of the contrast medium and the impossibility to obtain another venous access, failed to confirm the diagnosis via this imaging modality. In human medicine, a definitive gold standard for diagnosis remains elusive, with imaging techniques serving a critical role in generating diagnostic suspicion (13). The primary modalities utilized include ultrasound with Doppler assessment, followed by Magnetic Resonance Imaging (MRI), which is less accessible in veterinary practice. CT is used infrequently due to concerns regarding radiation exposure and the superior sensitivity of MRI in evaluating soft tissue structures (12). Ultimately, a definitive diagnosis is often achieved through the visualization of the twisted ovary during laparotomy (12).

In human medicine, where ovarian torsion typically presents with acute symptoms, surgical intervention is often performed promptly. The gold standard in such cases is to preserve the affected ovary by simply detorsing it. This technique boasts a high success rate, and most patients retain ovarian function following torsion (13). However, in instances where ovarian pathologies, such as tumors, are present, a unilateral oophorectomy is indicated. In the limited case reports involving avian and reptilian species, surgical intervention was performed in only a few instances, resulting in bilateral ovariosalpingectomy. In the present case, given the chronic nature of the torsion, the presence of preovulatory stasis and the owner’s lack of interest in maintaining the animal’s fertility, a bilateral ovariosalpingectomy was elected. A different approach may have been considered in other chelonian species of greater economic and conservation significance.

In human medicine, studies have shown that over 80% of patients undergoing surgery for ovarian torsion present with a mass at the ovarian level, typically an ovarian cyst or neoplasm, with a diameter greater than 5 cm (13). This finding is often correlated with the primary cause of ovarian torsion in humans. In contrast, the underlying causes of ovarian torsion in reptiles and avian species are not consistently identified. Some cases have reported the presence of concurrent infections affecting either the reproductive system or other organs, as well as signs of preovulatory follicular stasis in both ovaries (1–3). However, it remains unclear whether these factors directly contribute to ovarian torsion, as similar alterations have not been observed in all affected individuals. In our case, a concurrent bacterial coelomitis accompanied by preovulatory follicular stasis was noted. It is hypothesized that the presence of multiple large follicles may have induced a “mass effect,” mechanically leading to the torsion, as reported in human cases.

## Conclusion

Reptile medicine is constantly evolving, and it is crucial when working with these animals not to exclude unreported pathologies and to use advanced diagnostic tools to investigate issues thoroughly. In reptiles, cases of suspected preovulatory follicular stasis should not be limited to preliminary diagnosis or ultrasound examination. The use of diagnostic tools like Color Flow, or more advanced techniques like CT or MRI can aid in adding ovarian torsion to the differential diagnosis list.

## Data Availability

The original contributions presented in the study are included in the article/supplementary material, further inquiries can be directed to the corresponding author.
